# EVLTraining®: aplicativo para treino do cálculo da densidade de energia endovenosa linear

**DOI:** 10.1590/1677-5449.000816

**Published:** 2016

**Authors:** Alexandre Campos Moraes Amato, Salvador José de Toledo Arruda Amato

**Affiliations:** 1 Universidade de Santo Amaro – UNISA, São Paulo, SP, Brasil.; 2 Amato – Instituto de Medicina Avançada, São Paulo, SP, Brasil.

**Keywords:** varizes, validação de programas de computador, *software*, *lasers*

## Abstract

**Contexto:**

O reconhecimento da termoablação endovenosa como tratamento da insuficiência de veias safenas traz a necessidade de maior conhecimento e compreensão do método. A densidade de energia endovenosa linear (*linear endovenous energy density* – LEED) é a variável mais aceita para padronização, cada vez mais amplamente divulgada. Não deve ser utilizada apenas para comparação de resultados científicos – deve também ser usada no intraoperatório para a realização do procedimento com segurança.

**Objetivos:**

Desenvolver aplicativo para mensuração da LEED e avaliar sua aplicabilidade.

**Métodos:**

O aplicativo para iOS EVLTraining® foi desenvolvido para equipamentos portáteis com o intuito de padronizar a emissão energética em procedimentos de termoablação endovenosa.

**Resultados:**

O aplicativo criado mostrou equivalência com as medidas aplicadas no procedimento cirúrgico.

**Conclusões:**

O *software* EVLTraining® permite o treino prévio de cirurgião e equipe para a velocidade de tração da fibra ótica que será aplicada.

## INTRODUÇÃO

O reconhecimento da termoablação endovenosa como tratamento da insuficiência de veias safenas^1^ traz a necessidade de maior conhecimento e compreensão do método. Diferentemente da radiofrequência, que possui poucas variáveis de configuração do aparelho e cujo método é mais facilmente reprodutível, a termoablação endovenosa por *laser* requer o conhecimento do comprimento de onda, potência, energia, irradiância, fluência, tempo e distância, tanto para a escolha do equipamento apropriado como para a realização do procedimento de forma segura. O tipo de fibra ótica utilizada também influencia a quantidade de energia aplicada no tecido biológico. São conceitos da física transpostos para a medicina que necessitam ser amplamente elucidados. A densidade de energia endovenosa linear (*linear endovenous energy density* – LEED) é a variável mais aceita para padronização, cada vez mais amplamente divulgada. Não deve ser utilizada apenas na comparação de resultados científicos, mas também no intraoperatório para a realização do procedimento com segurança. Para aplicar a LEED, é necessário compreender a física e a matemática usadas na fórmula. Além disso, diferentemente do que se acredita, não é necessário fazer aproximações grosseiras ou técnicas matemáticas mentais difíceis, podendo ser facilmente calculada em tempo real.

Devido às diferenças entre os aparelhos disponíveis para a cirurgia de *laser*, com apresentação de dados diferentes, a padronização se faz necessária para aplicação da energia pretendida e comparação.

O *endolaser* venoso possui uma curva de aprendizado maior que outras técnicas exatamente devido à grande quantidade de variáveis que influenciam o resultado final. As múltiplas variáveis também dificultam a comparação entre trabalhos científicos. A alta variedade de equipamentos disponíveis, com grande variação nas informações apresentadas, pode confundir o cirurgião novato, que não saberá qual informação é mais importante para o melhor resultado final.

O treinamento prévio com modelos reais^2^ e virtuais tem se mostrado eficaz na simulação de procedimentos médicos com intuito educativo, e pode ser eficaz também na padronização da técnica.

Visamos elucidar o uso da LEED como treinamento para adquirir a competência necessária para realizar uma termoablação com êxito.

## MÉTODO

O aplicativo para iOS EVLTraining® (Amato, São Paulo, Brasil)^3^ foi desenvolvido em Objective-C para equipamentos portáteis com o intuito de padronizar a emissão energética em procedimentos de termoablação endovenosa. Entre os diversos parâmetros utilizados para mensuração da energia aplicada e também utilizados em trabalhos científicos, a LEED foi escolhida como padrão no *software* porque se mostrou adequadamente reprodutível. O método requer estratégia de uso e manipulação do *laser*, e possui elevada curva de aprendizado. A fixação de parâmetros, com alteração apenas da velocidade de tração, é o método mais reprodutível, porém requer treino e destreza. O aplicativo desenvolvido apresenta um meio virtual de cálculo da LEED, com livre escolha dos parâmetros utilizados, e permite o treino da velocidade de tração da fibra.

**Figura 1:**
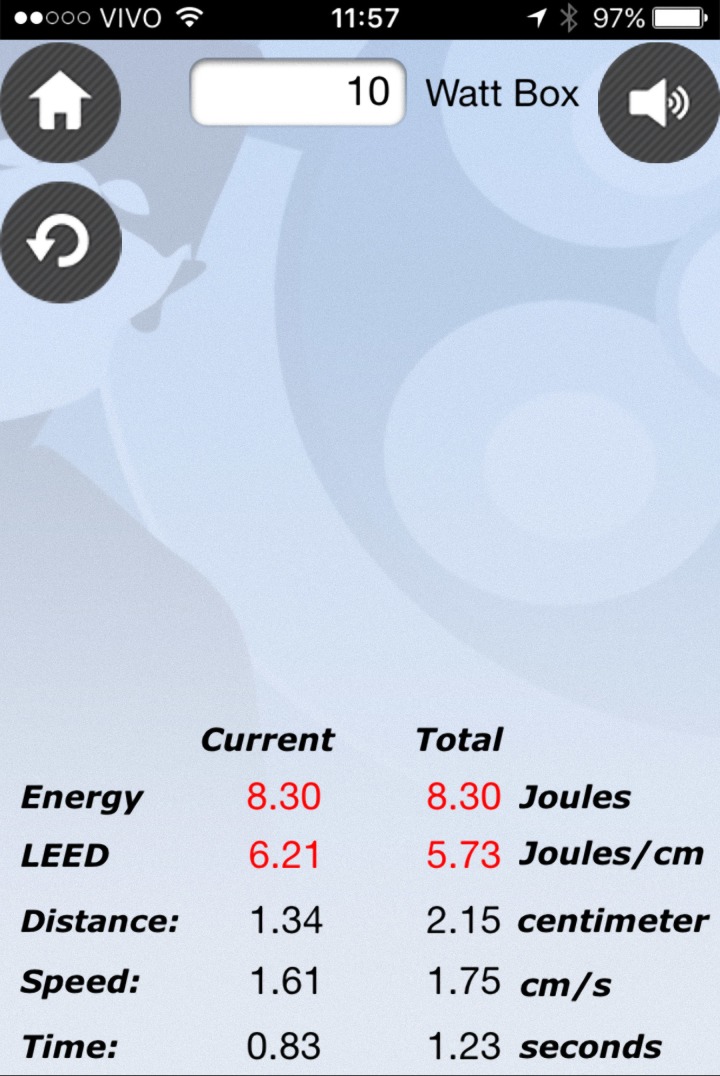
Janela principal do aplicativo EVLTraining® evidencia as variáveis controladas. A mesma tela é responsável pela medida pelo toque.

Para o teste do *software*, o equipamento iPhone® (Apple, Cupertino, Estados Unidos) foi envolto em plástico estéril e utilizado com o EVLTraining® no intraoperatório de termoablação endovenosa de safena. Ao tracionar a fibra ótica com o primeiro e o segundo dedos da mão dominante, estes deslizavam tocando sobre a tela capacitiva do celular, permitindo o cálculo em tempo real dos parâmetros aplicados. O procedimento cirúrgico foi gravado em vídeo para posterior análise. Os parâmetros evidenciados no *software* (Figura 1) foram comparados com os apresentados pelo equipamento de *laser* (energia parcial), relógio (tempo) e distância (fibra centimetrada).

## RESULTADOS

O aplicativo criado mostrou equivalência com as medidas aplicadas no procedimento cirúrgico. A velocidade de tração e a LEED comparadas posteriormente em vídeo foram equivalentes às do procedimento cirúrgico.

## DISCUSSÃO

O comprimento de onda é a distância entre valores repetidos sucessivos em um padrão de onda. No caso do *laser*, o comprimento de onda é determinado pela vibração térmica dos átomos, pela presença de impurezas no material que emite a luz, pela forma de energizá-lo e pelo sistema ótico aplicado. Normalmente, é um valor fixo, embora possa ser alterado com filtros específicos. Portanto, é determinado no momento da aquisição do equipamento de *laser*. Cada comprimento de onda apresenta um diferente cromóforo, ou seja, um tecido alvo de maior absorção^4^.

A energia é uma magnitude abstrata que está ligada ao estado dinâmico de um sistema fechado e que permanece invariável com o tempo. A energia térmica é, no fundo, energia cinética. Não se deve confundir energia térmica e temperatura, e também não se deve pensar que a temperatura é uma medida direta da energia térmica de um sistema. A energia térmica é medida em joules (J) no Sistema Internacional de Unidades (SI). Um joule compreende a quantidade de energia correspondente à aplicação de uma força de um newton durante um percurso de um metro. Ou seja, é a energia que acelera uma massa de 1 kg a 1 m/s^2^ em um espaço de 1 m^5^. 1J = 1kg x m^2^/s^2^. A energia é resultante da potência multiplicada pelo tempo de disparo (J=W*s), ou seja, o controle único da energia permite o controle de duas variáveis: tempo e potência^4^.

A potência é a grandeza que determina a quantidade de energia concedida por uma fonte a cada unidade de tempo. Em outros termos, potência é a rapidez com que uma certa quantidade de energia é transformada, ou é a rapidez com que o trabalho é realizado. Também pode ser entendida como a força multiplicada pela velocidade. O watt (W) é a unidade de potência do SI. É equivalente a um joule por segundo (1 J/s)^4^^,^ 5.

Portanto, a LEED consiste em potência x tempo / distância, ou W x s / cm. A medida da LEED, apesar de não ser perfeita, compreende quatro variáveis importantes para o *laser*: a potência, o tempo, a distância e, consequentemente, a energia. Sendo o comprimento de onda fixo, já são cinco variáveis controladas no procedimento. Com apenas uma variável, torna-se factível o controle intraoperatório em tempo real de cinco características importantes do *laser*.

A irradiância é sinônimo de densidade de potência, que é definida como a potência ótica útil do *laser*, expressa em W, dividida pela área irradiada, expressa em centímetros quadrados (cm^2^).

**Figura 2:**
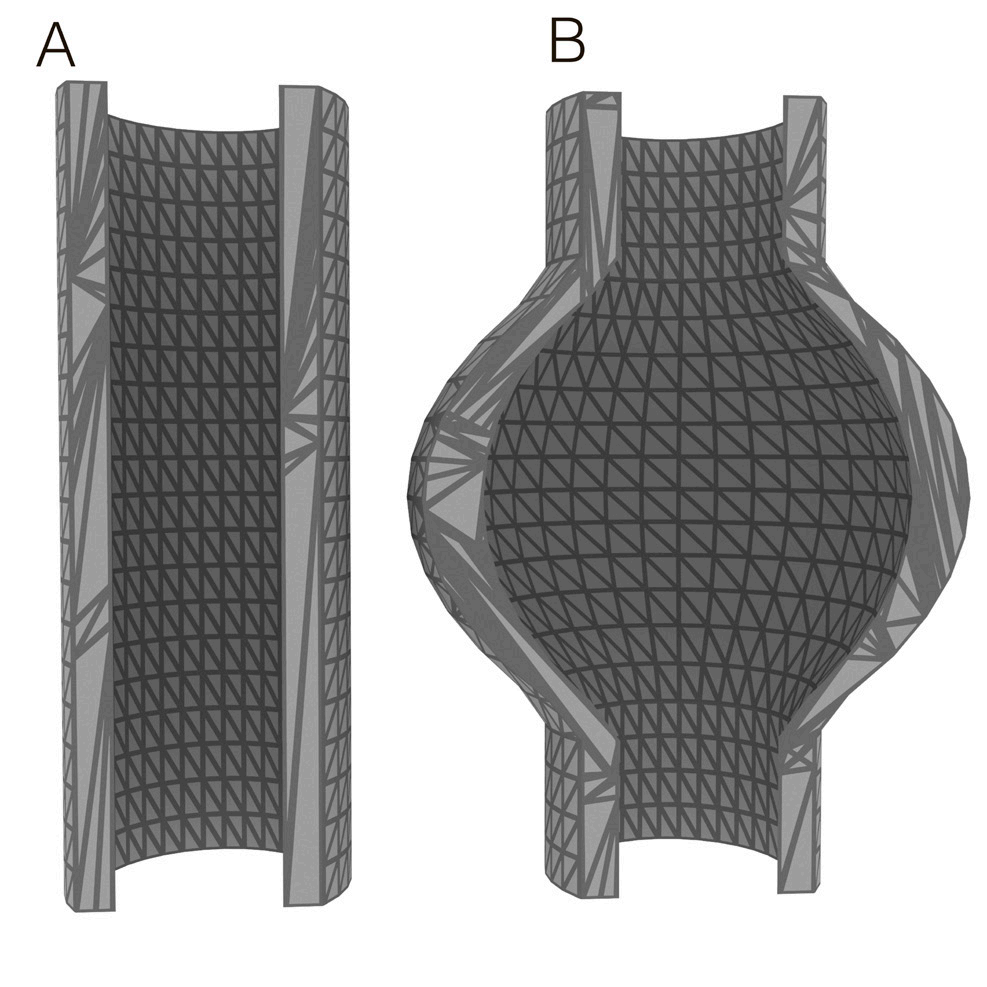
Modelo 3D de (A) veia cilíndrica e (B) veia dilatada evidencia o aumento da área de superfície interna na dilatação venosa.

A fluência é o termo utilizado para a taxa de energia que está sendo aplicada ao tecido. Ao multiplicar a irradiância pelo tempo de exposição (s), obtemos a fluência ou densidade de energia expressa em joules por centímetro quadrado (J/cm^2^)^6^. A fluência é mais utilizada como parâmetro para *laser* transdérmico por associar a área atingida pelo *laser*. O conceito de LEED é a variação da fluência, em que o denominador, em vez da área, é a distância linear, sendo então aplicável a regiões tubulares, como as veias. Obviamente, quando veias são mais dilatadas, a superfície da área interna cilíndrica é maior (Figura 2). Então, com a mesma LEED (que não considera a área), a densidade de energia aplicada ao tecido biológico é menor. Por isso, trabalhos recentes associam o diâmetro da veia a ser tratada com a LEED a ser aplicada^7^, aumentando conforme o aumento do diâmetro. Nesses casos, a fluência calculada com a área interna da veia seria a mesma: aumentando o denominador, que é a área, o nominador, que é a energia, deve aumentar para manter a fluência estável; mas ao utilizar a LEED como parâmetro, o aumento da área interna da veia não é contabilizado, sendo necessário compensar com o aumento da LEED aplicada.

Equipamentos mecânicos automatizados de tração permitem a fixação dos parâmetros e o cálculo prévio da energia que será aplicada ao paciente, mas apresenta desvantagens, como contaminação da fibra para reuso no mesmo procedimento e dificuldade de variação da LEED em veias com diâmetros diferentes.

Caso o equipamento de *laser* utilizado apresente ao usuário a variável energia parcial a cada disparo, considera-se que o aparelho esteja multiplicando o tempo do disparo (s) pela potência (W). Deve-se notar que o valor incrementa com o passar do tempo. Nesse caso, se for tracionado apenas um centímetro da fibra ótica, seguindo a fórmula apresentada, o denominador será 1 (um), a fórmula será facilmente calculável e o valor apresentado será equivalente à LEED. Disparos contínuos por distâncias maiores que um centímetro dificultam o cálculo mental da LEED, obrigando a observação da velocidade (cm/s).

O *software* EVLTraining® permite calcular velocidade de tração (cm/s), distância percorrida (cm), tempo (s), energia (J) e, consequentemente, a LEED (J/cm) para treinamento da equipe^8^. Quando o equipamento de *laser* utilizado não apresenta a energia parcial, ou quando a equipe não utiliza o método proposto de cálculo de LEED, é necessário realizar múltiplos cálculos mentais. Nesse caso, o treino prévio da velocidade de tração pelo cirurgião pode ser benéfico.

Entre as variáveis intraoperatórias, a potência (W), a energia (J) total, a energia parcial, o tempo (s) e a distância percorrida (cm) são as que exercem o controle sobre o procedimento.

A observação de todas as variáveis ao mesmo tempo dificulta o procedimento, aumentando desnecessariamente a complexidade.

## CONCLUSÃO

O *software* EVLTraining® permite o treino prévio de cirurgião e equipe para a velocidade de tração da fibra ótica que será aplicada.

## References

[1] Gloviczki P, Gloviczki ML (2012). Guidelines for the management of varicose veins. Phlebology.

[2] Amato ACM, Freitas SL, Veloso PM, Correia TCV, Santos RV, Amato SJTA (2015). Treinamento de punção ecoguiada em modelo de gelatina. J Vasc Bras.

[3] Amato Software (2012). EVLTraining.

[4] van den Bos RR, Kockaert MA, Neumann HA, Nijsten T (2008). Technical review of endovenous laser therapy for varicose veins. Eur J Vasc Endovasc Surg.

[5] Barrow GM (1982). Físico-química.

[6] Proebstle TM, Krummenauer F, Gül D, Knop J (2004). Nonocclusion and early reopening of the great saphenous vein after endovenous laser treatment is fluence dependent. Dermatol Surg.

[7] Maurins U, Rabe E, Pannier F (2009). Does laser power influence the results of endovenous laser ablation (EVLA) of incompetent saphenous veins with the 1 470-nm diode laser? A prospective randomized study comparing 15 and 25 W. Int Angiol.

[8] Mueller RL, Bridget M, Mueller J (2013). Digital metronomes and metric devices for venous ablation procedures. J Vasc Ultrasound..

