# Supplementary material for: EVLTraining®: aplicativo para treino do cálculo da densidade de energia endovenosa linear
Source: J Vasc Bras. 2016 Apr-Jun;15(2):134–7. [Article in Portuguese] doi: 10.1590/1677-5449.000816 (PMC5829707; doi:10.1590/1677-5449.000816)
Supplement: Translation

# Translation: EVLTraining®: mobile app for training and calculating linear endovenous energy density

**Authors:** Alexandre Campos Moraes Amato, Salvador José de Toledo Arruda Amato

**Affiliations:** 1 Universidade de Santo Amaro – UNISA, São Paulo, SP, Brazil.; 2 Amato – Instituto de Medicina Avançada, São Paulo, SP, Brazil.

**Keywords:** varicose veins, software validation, software, lasers

## Abstract

**Background:**

Recognition of endovenous thermal ablation as a treatment for saphenous vein insufficiency brings a need for greater knowledge and understanding of the method. Linear endovenous energy density (LEED) is the most accepted variable for standardization and it has been covered in a growing number of publications. However, it should not exclusively be used for comparison of scientific results, it should also be used intraoperatively so that the procedure is conducted safely.

**Objectives:**

To develop a mobile app for measurement of LEED and evaluate its applicability.

**Method:**

The application for iOS EVLTraining® was developed for portable devices in order to standardize energy emission in endovenous thermal ablation procedures.

**Results:**

The application developed demonstrated equivalence to measures applied during surgical procedures.

**Conclusion:**

The EVLTraining® software enables surgeons and staff to train the optical fiber pullback speed to be applied prior to surgery.

## INTRODUCTION

Recognition of endovenous thermal ablation as a treatment for saphenous vein insufficiency^1^ brings a need for greater knowledge and understanding of the method. In contrast with radio frequency ablation, in which the equipment has few variables to be configured and the method is more easily reproduced, endovenous thermal ablation by laser demands knowledge of wavelength, power, energy, irradiance, fluence, time and distance, when choosing equipment and while conducting the procedure safely. The type of optical fiber used also has an influence on the quantity of energy administered to biological tissues. These are concepts from physics transposed to medicine and they need to be thoroughly elucidated. Linear endovenous energy density (LEED) is the most accepted variable for standardization and it is covered in a growing number of publications. However, it should not be used exclusively for comparison of scientific results, it should also be used intraoperatively so that the procedure is conducted safely. In order to apply LEED, it is necessary to understand the physics and mathematics used in the formula. Additionally, in contrast with what is commonly believed, it is not necessary to make rough approximate estimates or use difficult mental arithmetic techniques, it can easily be calculated in real time.

Since there are differences between different types of equipment available for laser surgery, including differences in presentation of data, standardization is necessary so that the energy levels chosen can be compared.

Venous endolaser treatment has a greater learning curve than other techniques, exactly because of the large number of variables that influence the final result. These multiple variables also make it difficult to make comparisons between scientific studies. The great variety of equipment available and the great variation in the information presented could confuse inexperienced surgeons, who may not know which information is the most important to achieving the best final results.

Prior training with real and virtual models^2^ has proven effective for simulation of medical procedures for the purposes of education, and could also prove effective for standardization of the technique.

Our aim is to explain how LEED can be used in training for acquisition of the competence necessary to conduct successful thermal ablation.

## METHOD

The EVLTraining® app for iOS (Amato, São Paulo, Brazil)^3^ was developed in Objective-C for portable devices with the objective of standardizing energy emission during endovenous thermal ablation procedures. From among the many parameters for measurement of energy levels that are employed in practice and in scientific studies, LEED was chosen as the software’s standard because it is sufficiently reproducible. This method is dependent on the strategy for use and handling of the laser and the learning curve involved is accentuated. The most reproducible method is to set all parameters except pull-back velocity at fixed levels, but it demands training and dexterity. The app offers a virtual environment for calculating LEED, with a free choice of the parameters to be used, and allows the user to practice the fiber pull-back velocity.

For the software test, an iPhone® (Apple, Cupertino, United States) device was wrapped in sterile plastic and used to run EVLTraining® during a saphenous endovenous thermal ablation procedure. As the operator pulled the optical fiber back with the first and second fingers of the dominant hand, these slid along the capacitive screen of the device, allowing it to calculate the parameters applied in real time. The surgical procedure was video-recorded for later analysis. The parameters shown by the software (Figure 1) were compared with those registered on the laser equipment (partial energy), clock (time) and graded fiber (distance in centimeters).

**Figure 1:** Main window of the EVLTraining® app showing the variables controlled. The same screen is responsible for measurement by touch.

## RESULTS

The app demonstrated equivalence to the measures used during the surgical procedure. The pull-back velocity and LEED compared on the video were equivalent to those employed during the surgical procedure.

## DISCUSSION

Wavelength is the distance between successive repeated values in a waveform. In the case of lasers, the wavelength is determined by the thermal vibration of atoms, by the presence of impurities in the material that emits the light, by the way it is energized and by the optical system applied. It is normally a fixed value, although it can be altered with specific filters. It is therefore determined at the time at which the laser equipment is purchased. Each wavelength has a different chromophore, i.e., a target tissue that exhibits greatest absorption.^4^

Energy is an abstract magnitude that is linked to the dynamic state of a closed system and remains unchanged over time. Thermal energy is, fundamentally, kinetic energy. Thermal energy must not be confused with temperature and it is also a mistake to think that temperature is a direct measurement of the thermal energy of a system. Thermal energy is measured in joules (J) within the International System of Units (SI). One joule is the quantity of energy equivalent to a 1 Newton force applied through a distance of 1 meter. In other words, it is the energy needed to accelerate a mass of 1 kg at 1 m/s^2^ through a 1 m distance in space.^5^ 1J = 1kg x m^2^/s^2^. Energy is the result of power multiplied by the pulse duration (J=W*s), so control of energy allows two variables to be controlled: time and power.^4^

Power is the magnitude that determines the quantity of energy delivered by a source for each unit of time. In other terms, power is the speed with which a certain quantity of energy is transformed, or it is the speed with which work is performed. It can also be understood as force multiplied by velocity. The SI unit of power is the watt (W). It is equivalent to 1 joule per second (1 J/s).^4^^,^ 5

Therefore, LEED consists of power x time / distance, or W x s / cm. Measuring LEED, although imperfect, takes in four important variables of laser treatment: power, time, distance and, consequently, energy. Since wavelength is fixed, this amounts to control over five of the variables in the procedure. Using just a single variable, it becomes possible to have real-time intraoperative control over five important characteristics of the laser treatment.

Irradiance is a synonym of power density, which is defined as the usable optical power of the laser, expressed in W, divided by the area irradiated, expressed in square centimeters (cm^2^).

Fluence is a term used to describe the proportion of energy that is being administered to the tissues. Multiplying irradiance by exposure time (s), gives us the fluence or energy density, expressed in joules per square centimeter (J/cm^2^).^6^ Fluence is most used as a parameter for transcutaneous lasers since it is associated with the area reached by the laser. The concept underlying LEED is the variation in fluence, in which the denominator, rather than being area, is linear distance, making it applicable to tubular structures, such as veins. Obviously, when veins are more dilated, the internal cylindrical surface area is greater (Figure 2). Thus, the same LEED (which does not consider area) will result in lower energy density applied to the biological tissue. For this reason, more recent studies relate the diameter of the vein to be treated to the LEED to be administered,^7^ which increases as the diameter increases. In this case, the fluence calculated using the internal area of the vein will be the same: when the denominator, which is the area, increases, the numerator, which is energy, must also increase to keep fluence stable. However, when LEED alone is used as the parameter, increases in the internal vein area are not calculated and must be compensated for by increasing the LEED applied.

**Figure 2:** 3D models of (A) cylindrical vein and (B) dilated vein, showing the increase in internal surface area of the dilated vein.

Automated mechanical pull-back equipment makes it possible to set the parameters and calculate the energy administered to the patient in advance, but there are disadvantages, such as contamination of a fiber that would be reused in the same procedure and difficulties with varying the LEED in veins with different diameters.

If the laser equipment employed displays to the user the variable partial energy per pulse, it is understood that the machine is multiplying the duration of the pulse (s) by power (W). It should be noted that this value increases as time passes. In this case, according to the formula, if just 1 cm of the optical fiber is pulled back, then the denominator will be 1, the equation will be easy to calculate and the resulting value will be equivalent to the LEED. Continuous pulses over distances greater than 1 centimeter make it more difficult to calculate LEED using mental arithmetic, and so it becomes necessary to observe the velocity (cm/s).

The EVLTraining® software offers the possibility of calculating the pull-back velocity (cm/s), distance pulled-back (cm), time (s), and energy (J) and, consequently, LEED (J/cm) for training the surgical team.^8^ When the laser equipment employed displays the partial energy, or when the team does not use the proposed method for calculation of LEED, it is necessary to perform multiple mental calculations. In this case, prior training of the surgeon in pull-back velocity should be beneficial.

Of the intraoperative variables, it is power (W), total energy (J), partial energy, time (s) and distance pulled-back (cm) that control the procedure.

Observation of all of these variables at the same time makes the procedure more difficult, unnecessarily increasing its complexity.

## CONCLUSIONS

The EVLTraining® software enables surgeons and staff to train the optical fiber pullback speed to be applied prior to surgery.
